# Synaptic Transistors Exhibiting Gate-Pulse-Driven, Metal-Semiconductor Transition of Conduction

**DOI:** 10.3390/ma14247508

**Published:** 2021-12-07

**Authors:** Jung Wook Lim, Su Jae Heo, Min A. Park, Jieun Kim

**Affiliations:** 1 Information & Communications Core Technology Creative Research Laboratory, Electronics and Telecommunications Research Institute (ETRI), 218 Gajeong-ro, Daejeon 305-700, Korea; sjheo21@etri.re.kr (S.J.H.); parkmina88@etri.re.kr (M.A.P.); lolila@etri.re.kr (J.K.); 2 Department of Advanced Device Engineering, University of Science and Technology (UST), 217 Gajeong-ro, Daejeon 305-350, Korea

**Keywords:** synaptic device, metal-semiconductor transition, linear excitatory behavior, charge inducing dielectric

## Abstract

Neuromorphic devices have been investigated extensively for technological breakthroughs that could eventually replace conventional semiconductor devices. In contrast to other neuromorphic devices, the device proposed in this paper utilizes deep trap interfaces between the channel layer and the charge-inducing dielectrics (CID). The device was fabricated using in-situ atomic layer deposition (ALD) for the sequential deposition of the CID and oxide semiconductors. Upon the application of a gate bias pulse, an abrupt change in conducting states was observed in the device from the semiconductor to the metal. Additionally, numerous intermediate states could be implemented based on the number of cycles. Furthermore, each state persisted for 10,000 s after the gate pulses were removed, demonstrating excellent synaptic properties of the long-term memory. Moreover, the variation of drain current with cycle number demonstrates the device’s excellent linearity and symmetry for excitatory and inhibitory behaviors when prepared on a glass substrate intended for transparent devices. The results, therefore, suggest that such unique synaptic devices with extremely stable and superior properties could replace conventional semiconducting devices in the future.

## 1. Introduction

Existing semiconductor devices face diverse challenges as a result of continuous downscaling, emphasizing the need for technological breakthroughs to address these problems [1,2,3]. One promising solution is the development of smart devices capable of simultaneously accommodating multiple states, which can be easily implemented using simple stimuli, such as voltage or light pulses. In particular, these devices are essential for the implementation of neuromorphic devices such as artificial synapses and neurons, as well as ultrafast switches and various sensors [4,5]. To ensure the successful commercialization of such devices, their multiple states must be distinguished by a sufficiently large margin of electrical conductivity, and their current must be linearly and symmetrically potentiated and depressed by voltage pulses. More importantly, each state must have long-term stability and reproducibility. Furthermore, these smart devices must overcome the von Neumann bottleneck, a term that refers to the significant gap between processors and memory [6].

A voltage pulse should trigger an excitatory current in neural systems that lasts until the next pulse is applied [7]. Due to their low power consumption, simple structure, and small cell size, two-terminal devices have been explored to achieve human-brain-like computers. However, they suffer from critical drawbacks such as device variability and operation instability, which hinder their application in advanced artificial intelligence systems [8]. As a result, three-terminal devices have been intensively studied in recent years as promising alternatives, due to their high stability and simple operation mechanism [9,10,11]. However, transistor-based devices use floating, ferroelectric, and electrolyte gates for accumulating charges [10,11]. As a result, the amplification of conductivity is significantly lower than the required level in these human-brain-like devices, in addition to their complex structure and poor reproducibility [8]. Despite the half success of the three-terminal devices, they are not satisfactory in terms of linearity for high learning accuracy, and there is still considerable scope for improvement [9,10,11]. In addition, there is an urgent need to develop synaptic devices that achieve excellent symmetry in both potentiation and depression processes during the learning process.

The device proposed in this study utilizes a nanoscale oxide channel layer and is based on the trap-and-release mechanism of charged carriers in three-terminal devices with the unique property of gate-pulse-driven, metal-semiconductor transition (MST). Additionally, it exhibits higher stability and a much larger on/off ratio (>10^7^), in comparison to conventional Mott metal-insulator transition (MIT) devices. Although two-terminal devices based on the existing Mott MIT phenomenon have been reported, their versatility and stability have been largely limited [12]. The proposed three-terminal device, however, shows significant changes in current levels when the gate voltage spikes are supplied. In three-terminal devices, the semiconductor state is in the “off” state, while the metallic state is in the “on” state. The MST can be controlled by merely supplying a gate voltage pulse, resulting in abrupt changes in conductivity or mobility. Thus, they make excellent candidates for ultrafast switches, actuators, and neuromorphic devices [13,14]. The majority of the studies on MIT materials have focused on changes in conductivity that are temperature dependent [15,16,17]. However, such MIT materials are not applicable to practical electronic devices, such as neuromorphic devices and multi-level memory devices, which are conventionally operated by voltage or current pulses. For instance, VO_2_ is a representative MIT material, whose transitions have been observed under an applied voltage [18,19]. Although two-terminal devices can be operated based on the changes in resistivity caused by the phase transition of VO_2_, they are rendered thermally unstable with a transition temperature of <70 °C. As a result, techniques for the implementation of MIT devices, such as a three-terminal transistor with a more stable material, are required for the stable operation, accurate current level control, and compatibility with conventional Si-based devices.

Unlike MIT devices reported previously, merely supplying a voltage pulse to the gate imparts MST characteristics to the proposed three-terminal device. In addition to the pulse intensity, the number of trapped charges at the interfaces can also be effectively modulated by controlling the pulse width and number of voltage pulses. Furthermore, the conductance of the channel layers can be altered significantly using deep traps. Additionally, the gate oxide films function as the dielectric layer and the charge-inducing dielectric (CID), resulting in a substantial increase in the number of carriers in the channel layer. The deep traps capture excess carriers within the CID, while the charged trap sites induce numerous carriers of the opposite polarity in the channel layer, resulting in abrupt changes in the conduction state of the channel layer. In our previous studies, we proposed oxide-FET-based, non-volatile memory devices with highly reproducible memory operations [20,21]. The devices proposed in the current study also exhibit a shift in the threshold voltage with respect to the incident optical pulse, and offer the selective removal of shallow traps by H_2_ plasma treatment, which improves their operation stability [20,22]. Furthermore, we leverage the MST properties for the fabrication of a transparent device that performs synaptic behaviors. Additionally, the devices were expanded to transparent synaptic transistors to demonstrate the long-term plasticity (LTP) and learning behaviors, including potentiation and depression curves with excellent linearity and symmetry.

## 2. Materials and Methods

For the fabrication of bottom-gate, metal-oxide-semiconductor, field-effect transistors (MOSFETs), 80-nm-thick SiO_2+δ_ CID and 30-nm-thick TiO_2_ channel layers were sequentially deposited via Plasma Enhanced Atomic Layer Deposition (PEALD) at 200 °C on a low-resistivity (<0.005 Ω·cm), n-type Si wafer substrate that also served as the back-gate electrode. The TiO_2_ channel areas were patterned by combining photolithography and reactive ion etching. Additionally, the Al electrodes for the source and drain were deposited at room temperature using a thermal evaporation method and then defined using a lift-off process. The channel length and width were maintained at 30 and 40 μm, respectively, in the bottom-gate MOSFET structure. The fabrication of the transparent devices, ZnO:Ga films (the content of Ga is 5 wt%), herein used as the gate electrode, were grown on glass substrates using a sputtering method. Additionally, the chemical bonding and composition were analyzed via electron spectroscopy (Thermo Electron Corporation, Waltham, MA, USA: Escalab200R), and the electrical characteristics of the fabricated MOSFETs were determined using a parameter analyzer (Keithley 4200, Solon, Ohio, OH, USA) in the dark at 25 °C.

## 3. Results & Discussions

### 3.1. Conduction States and Structures

In a conventional three-terminal transistor, when the doping concentration of the semiconductor channel is significantly high, no change in the drain current (*I_D_*) is observable with the variation in gate voltage. Converesly, a constant high conductance that is invariant to the gate voltage, i.e., a metallic state, is observed. However, as the doping concentration decreases, *I_D_* is modulated by the gate voltage, exhibiting typical switching properties of the semiconducting state. A device in which the metallic and semiconducting states can be reversibly controlled via external stimuli such as voltage pulses can function as an artificial intelligence device with synaptic plasticity [10]. However, few studies have investigated the implementation of such a smart device, capable of simultaneously representing the metallic and the semiconducting state. As a result, the current study explores a smart transistor in which the metallic and semiconducting states can be easily switched by reversing the polarity of the gate voltage pulses.

**Figure 1:**
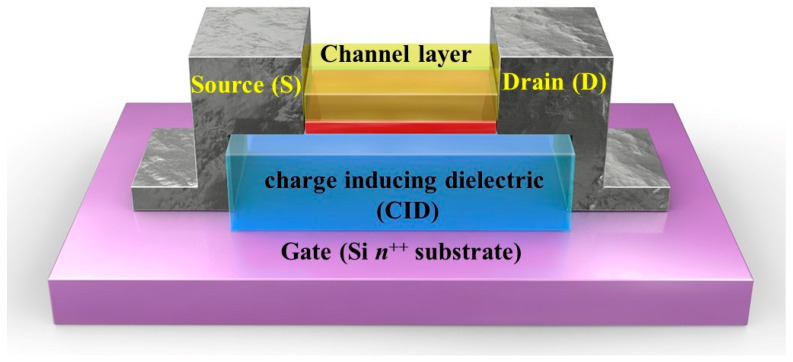
Schematic of a three-terminal device with a metal semiconductor transition (MST) operating principle. The device comprised a gate, source/drain electrodes, TiO_2_ channel layer, and charge-inducing dielectric (CID).

A schematic of the proposed three-terminal device, comprising a gate, source/drain electrodes, TiO_2_ channel layer, and CID, that inherently functions as a gate insulator, is illustrated in Figure 1. As reported previously, the trap states were located at the interface between the channel layer and the CID [20,21,22]. In this study, SiO_2+δ_ grown by plasma-enhanced atomic layer deposition (PEALD) was used as the CID. The composition analysis was carried out via X-ray photoelectron spectroscopy (XPS) (data not shown). As observed from the analysis, the composition ratio (O/Si) of the PEALD-SiO_2+δ_ films was marginally higher than that of thermally grown SiO_2_ films, synthesized before and after Ar^+^ ion sputter cleaning of the surface. The δ value of the oxygen content of the CID should remain ≈ 0.10 due to the highly stoichiometric nature of the thermally grown SiO_2_ films. As a result, the CID included a metal deficient structure and H-atoms as the impurity, which resulted in the formation of free holes in the oxide to satisfy charge neutrality. When free holes approached the traps located at the interfaces, they were trapped when a positive voltage pulse was applied to the gate. Owing to the Coulomb interaction between the carriers in the channel and the positively charged traps, the carrier concentration of the channel could be increased or decreased repeatedly and significantly by regulating the trap or by the release of the holes in the trap states using the gate voltage. This phenomenon appeared to be similar to ionic gating. However, the trapped charge induced carriers and persisted for an extended period of time due to the presence of trapped carriers in deep traps, even after the gate voltage pulse was removed. Unlike previously reported devices, MSTs occurred in this device [23].

### 3.2. Characteristics of MST Devices

**Figure 2:**
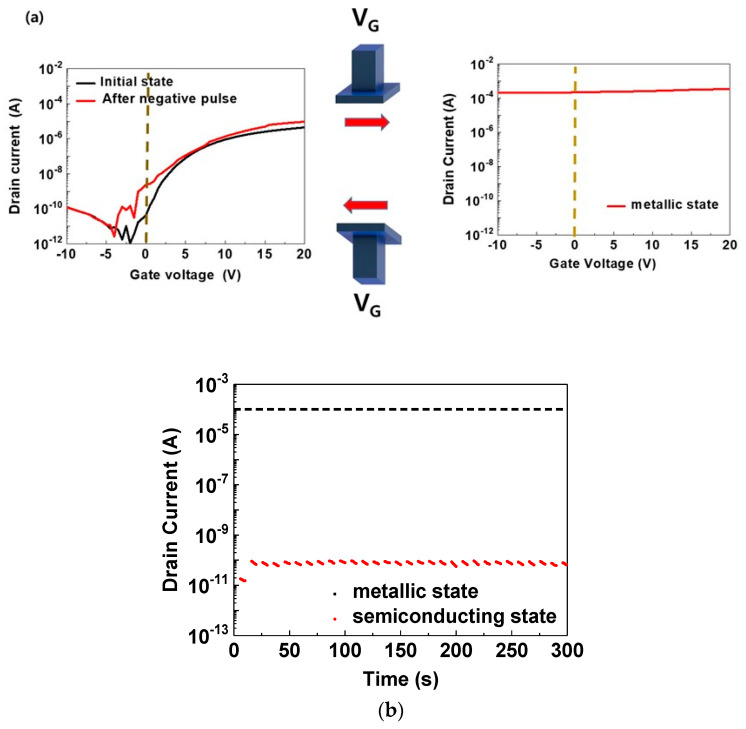
Characteristics of MST devices employing a CID with W/L = 40/30 μm fabricated on Si (*n^++^*) substrates. (**a**) Transfer curves of semiconducting and metallic states and gate voltage pulses for state conversion. (**b**) Variation in drain current for repeated switching of gate voltage polarity at *V_G_* = 0 V.

Figure 2a illustrates the transfer curves of the proposed MST devices fabricated on Si (*n^++^*) substrates with a width (W) and length (L) of 40 and 30 μm, respectively. The typical transfer curve of the semiconducting state was obtained as an initial state, and the *V_ds_* was fixed to 1 V. The mobility and sub-threshold voltage of the device was 4.89 cm^2^V^−1^s^−1^ and 1.44 Vdec^−1^, respectively. The mobility value of MST devices was observed to be relatively higher than TiO_2_-based transistors, reported previously [24,25,26]. Moreover, the on/off ratio exceeded a value of 10^6^ [7], which suggested that high-performance transistors can be manufactured using the PEALD SiO_2.1_ as a gate dielectric. Additionally, immediately within 0.1 s after a positive voltage pulse was applied to the gate (+30 V amplitude for 10 s), the number of carriers abruptly increased, converting the channel layer into a metallic state, as illustrated in the right panel of Figure 2a. For transistors based on oxides containing In, the switching behavior could not be accomplished because *I_D_* was maintained at a high constant value (i.e., a metallic state) owing to the high carrier density that originated from excessive defects, regardless of the magnitude of the gate voltage [27,28]. In such cases, it was impossible to convert the channel layer from the metallic state to the semiconducting state by external stimuli such as voltage and light pulses. Conversely, the state conversion during operation could be achieved in conventional devices via thermal treatment (Joule heating) to change the material phase. However, when a positive voltage pulse was applied to the gate of the MST devices fabricated in this study, the holes in the CID were drawn into the interface, captured by the interface’s trap site, and then positively charged to attract the electrons in the TiO_2_ channel layer toward the interface. Conversely, when a negative voltage pulse was applied to the gate, the metallic state returned to the semiconducting state owing to the de-trapped holes at the interfaces, as shown in the left panel of Figure 2a. To describe the mechanism in more detail, the films were analyzed using XPS and AES methods. The composition of CID was confirmed to have an oxygen excess. It is well known that such an oxygen excess creates excessive holes to achieve charge neutrality. When a positive gate voltage is applied, the holes inside the CID move toward the interface and are trapped by the trap sites of the interface, thereby inducing electrons in the channel and increasing the channel current. These charge traps can be identified by observing the hysteresis of the transfer curve, which has been reported in our previous works as well [20,21,22]. Therefore, the conductivity of the channel increases rapidly, which causes a change in the conduction phase. Conversely, when a negative gate voltage is applied, the trapped holes are released again, and the current returns to the initial states. Thus, novel device characteristics such as reversible behavior can be realized by repeatedly switching both states at *V_G_* = 0 V, as illustrated in Figure 2b. This ensures the reliable switching of currents with an on/off ratio exceeding 10^6^ by merely using voltage pulses.

### 3.3. Long Term Stability of Multi-States

**Figure 3:**
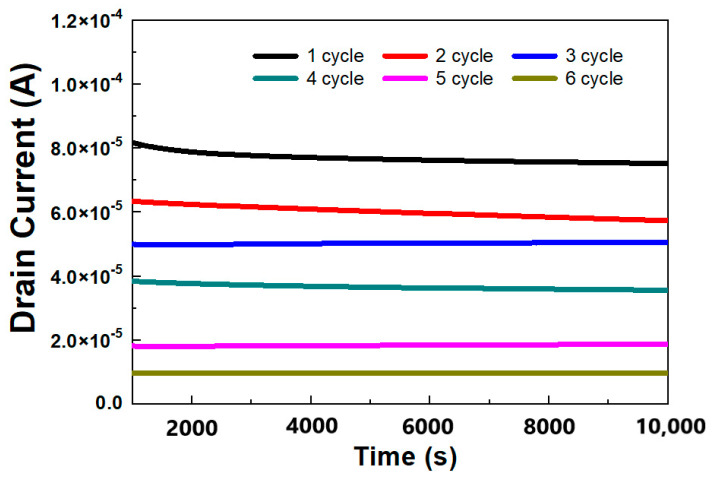
Variation in drain current for 10,000 s. From the metallic state, multiple states were formed by several negative pulses with each state maintaining a constant value under a drain voltage of 1 V by fixing *V_G_* to 0 V.

Another desirable feature of neuromorphic devices is long-term stability, which is critical to the implementation of logic operations and memory on a single device. To confirm this, the variation in *I_D_* over time was determined for each state. The durability of the multiple states created separately by several negative pulses applied to the device in the metallic state is illustrated in Figure 3. The multiple states that were formed during the transition from the metallic state to the semiconducting state were reproducibly controlled by several voltage pulses. After various gate voltage pulses were applied, the *I_D_* levels at *V_G_* = 0 V were maintained under a constant drain voltage (*V_D_*) of 1 V. Subsequently, each level retained its state without overlapping or crossing after 10,000 s, as shown in Figure 3. The results indicated that a long lifetime may be necessary to ensure the long-term stability of the as-fabricated MST devices. Furthermore, this characteristic is highly encouraging in that it effectively implemented the synaptic devices’ long-term plasticity.

### 3.4. Synaptic Behaviors of Transistors Fabricated on Glass Wafers

**Figure 4:**
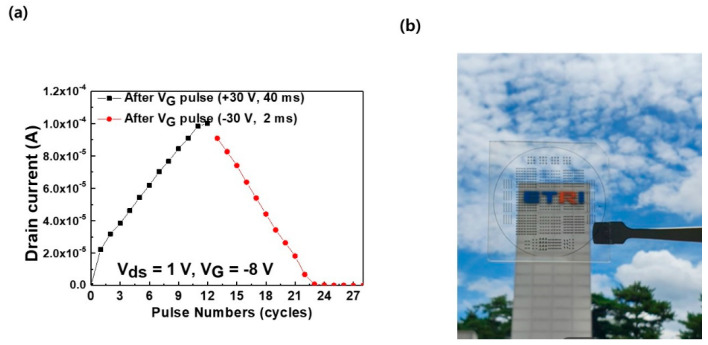
Devices fabricated on a glass substrate and connected in series and parallel, with transparent films of Ga-doped ZnO employed as the gate electrode. (**a**) Variation in drain current with number of pulses, exhibiting a linear and a symmetric relationship of excitatory and inhibitory behaviors, and (**b**) photograph of transparent devices except opaque Al metal electrodes.

The use of the proposed transistor provided a facile route for the fabrication of a device to perform basic synaptic behaviors of excitatory and inhibitory processes. The devices were constructed on a glass substrate and exhibited a high degree of transparency owing to the highly transparent channel and dielectric layers. Additionally, the Ga-doped ZnO (ZnO:Ga), a transparent electrode, was used as the bottom-gate electrode. As a result, the transmittance of visible light could be easily improved using ZnO:Ga as the source/drain electrode material. Because the polarity of the gate voltage pulses determines whether the charge is trapped or released, the device could be compared to the properties of a synaptic device, where potentiation and depression occur as a result of a gradual change in current caused by an external stimulus. When a positive pulse train is applied, followed by a negative pulse train, multiple conduction states can be obtained, resulting in excitatory and inhibitory synaptic behavior with excellent linearity (Figure 4a). Each positive pulse in the potentiation process had a pulse time of 40 ms, while the negative pulse train had a pulse time of 2 ms to satisfy symmetry as well as linearity. In the case of increasing durations, the linearity and symmetry became worse than those in current optimized conditions despite obtaining a higher drain current. The curves of potentiation and depression thereby show excellent symmetric behavior that was desired to achieve a high learning accuracy [8]. Additionally, the process successfully resulted in the formation of 12 states for each potentiation and depression while maintaining excellent linearity and symmetry. Moreover, the device exhibited very good reproducibility of synaptic behaviors. The resultant transparent devices are depicted in Figure 4b, where only the bottom gate employed transparent electrode while the source and drain used opaque Al electrodes. In the future, we plan to fabricate devices by replacing all electrodes with transparent electrodes, thereby fabricating fully transparent devices.

## 4. Conclusions

The current study details the fabrication of a novel three-terminal device comprising a gate, source/drain electrodes, TiO_2_ channel layer, and a unique CID, for use as a potential alternative to conventional semiconductors. The interface trap sites captured or released carriers supplied from the CIL, while the trapped charge density demonstrated a strong effect on the conducting behavior of the channel layers. Initially, the device exhibited a semiconducting behavior with a mobility and sub-threshold voltage of 6.83 cm^2^V^−1^s^−1^ and 1.15 V dec^−1^, respectively. However, upon the application of a positive voltage pulse to the gate, the semiconductor state transitioned into a metallic state, exhibiting a high, constant current, regardless of the gate voltage. Conversely, for the negative gate voltage pulse, the transition was observed to undergo a reversion. Thus, a reversible and reproducible transition between the metal and semiconductor states could be achieved, depending on the polarity of the gate pulse. Additionally, multiple levels were implemented during the transition from the metallic to the semiconducting state that were found to be distinct from each other. Furthermore, when the value of *V_G_* was kept constant, the multiple states were well maintained for 10,000 s, demonstrating excellent, long-term stability. Additionally, when a positive pulse train followed by a negative pulse train was applied to the gate, the synaptic behaviors of the excitatory and inhibitory current variation were observed to be highly linear and symmetrical in the transparent devices that were fabricated. These findings, therefore, pave the way for the development of synaptic devices capable of long-term plasticity.

## Data Availability

Data sharing not applicable.
